# Superdiffusive motion of membrane-targeting C2 domains

**DOI:** 10.1038/srep17721

**Published:** 2015-12-07

**Authors:** Grace Campagnola, Kanti Nepal, Bryce W. Schroder, Olve B. Peersen, Diego Krapf

**Affiliations:** 1 Department of Biochemistry and Molecular Biology, Colorado State University, Fort Collins, CO 80523, USA; 2 School of Biomedical Engineering, Colorado State University, Fort Collins, CO 80523, USA; 3 Department of Electrical and Computer Engineering, Colorado State University, Fort Collins, CO 80523, USA

## Abstract

Membrane-targeting domains play crucial roles in the recruitment of signalling molecules to the plasma membrane. For most peripheral proteins, the protein-to-membrane interaction is transient. After proteins dissociate from the membrane they have been observed to rebind following brief excursions in the bulk solution. Such membrane hops can have broad implications for the efficiency of reactions on membranes. We study the diffusion of membrane-targeting C2 domains using single-molecule tracking in supported lipid bilayers. The ensemble-averaged mean square displacement (MSD) exhibits superdiffusive behaviour. However, traditional time-averaged MSD analysis of individual trajectories remains linear and does not reveal superdiffusion. Our observations are explained in terms of bulk excursions that introduce jumps with a heavy-tail distribution. These hopping events allow proteins to explore large areas in a short time. The experimental results are shown to be consistent with analytical models of bulk-mediated diffusion and numerical simulations.

A myriad of signalling proteins are recruited to specific cell membranes via phospholipid-binding domains^1^,^2^. These molecules dock to the surface of specific lipid membranes and undergo two-dimensional diffusion in search of a target. Once the target is located, many proteins either activate or suppress a downstream signalling pathway for various physiological and pathological processes. Examples of membrane-targeting domains include pleckstrin homology (PH)^3^ and C2^4^, which have been identified in hundreds of human signalling molecules as well as in eukaryotic species as diverse as fungi and flies^5^. PH domains bind specifically to phosphoinositides while C2 domains bind a variety of membranes, and a subset of C2 domains only bind membranes in the presence of calcium and play key roles in signalling pathways. The association to lipid membranes often takes place in response to different extracellular and intracellular stimuli, but typically the residence on the membrane surface is only temporary. The transient nature of peripheral protein-membrane interactions enables a tight temporal regulation of signal transduction. Further, membrane dissociation has also broad implications on the search for the target substrate, but this process is less well-understood.

Recently, Knight and Falke observed the dissociation of PH domains from supported bilayers followed by rapid rebinding to the surface after a short excursion in the bulk solution^6^. They proposed that the hopping process may be important in the search for target molecules in eukaryotic cells. Subsequently, Yasui *et al.* found that PTEN (phosphatase and tensin homologue) molecules hop along the plasma membrane of living cells due to dissociation followed by rebinding^7^. PTEN is an important protein that suppresses development of cancer, where it prevents cells from growing and dividing too rapidly by dephosphorylating phosphoinositide substrates on the plasma membrane. PTEN-membrane affinity is regulated by a C2 domain and it is enhanced by electrostatic interactions^7^,^8^,^9^. The observed hopping of the C2 domain on the plasma membrane is thus expected to alter the dynamics of the search for a phospholipid substrate.

A straightforward consequence of membrane hopping is that a molecule remains in its immediate vicinity for a short time and then jumps to a location that is further away than expected from two-dimensional diffusion. Therefore, the search process is allowed to explore larger areas and the molecule can bypass diffusion barriers that may be present in the membrane. However, hopping comes at the cost of the search being less exhaustive. We may ask the questions how the dynamics of membrane-targeting domains is affected by such long jumps and how this motion deviates from simpler two-dimensional diffusion. Such potential complex behaviour can yield anomalous diffusion of membrane-targeting domains, which would alter the outcome of search processes and the sequential molecular reactions.

Anomalous diffusion is widespread in the motion of molecules in biological systems^10^,^11^,^12^,^13^. In general, a particle exhibits anomalous diffusion when the mean square displacement (MSD) scales as a power law with an exponent *α* ≠ 1


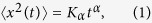


where *K*_*α*_ is the generalized diffusion coefficient with units cm^2^/s^*α*^. When *α* < 1 the process is subdiffusive and when *α* > 1 it is superdiffusive. Subdiffusion in the cytoplasm^14^,^15^,^16^, the nucleus^17^, and the plasma membrane^18^,^19^,^20^ of live cells is caused by crowding^21^,^22^ and complex interactions with the cytoskeleton and macromolecular complexes, among others. Similarly, subdiffusion can take place in model membranes due to crowding and packing effects^23^,^24^. The appearance of superdiffusion processes in biomolecular systems is far less common. The archetypal mode of superdiffusive motion is due to active cytoplasmic flows and transport mediated by molecular motors, requiring ATP energy consumption^25^,^26^,^27^.

From a theoretical point, there are three major mechanisms that can introduce superdiffusion^28^. It can be caused by correlations in the random walk, such as those in fractional Brownian motion with a Hurst index *H* > 1/2, by persistent directional motions (Lévy walks), and by long jumps (Lévy flights)^29^,^30^. Active biological transport can be modelled as Lévy walks^27^. Bulk-mediated diffusion processes, which can be described as Lévy flights, have been observed for transient adsorption on a solid surface where molecules display intermittent behaviour, alternating between periods of immobilization at the solid-liquid interface and periods of diffusion in the bulk fluid^31^,^32^.

In this article we report the experimental observation of superdiffusive transport of membrane-targeting C2 domains on supported lipid bilayers. Measurements of the diffusion of membrane-targeting domains are performed by single-particle tracking and are compared to both analytical theory and numerical simulations. In stark contrast to active cytoplasmic transport, superdiffusion on model membranes does not require energy. Our data strongly suggests that superdiffusion is caused by bulk-mediated diffusion, namely molecules dissociate from the membrane and perform three-dimensional random walks until they reach the membrane again and readsorb at a new location, as sketched in Fig. 1. Interestingly, the motion of membrane-targeting domains shows weak ergodicity breaking, a phenomenon that has recently attracted considerable attention in cellular environments and other complex systems^10^,^12^,^33^,^34^,^35^. The ergodic hypothesis, which is fundamental to statistical mechanics, states that ensemble averages and long-time averages of individual trajectories are equivalent. The violation of ergodicity has pronounced implications for the dynamics of individual molecules, which can be very different from the ensemble statistics^10^. In the traditional way of obtaining the MSD, the square displacements are averaged over a large ensemble of molecules at a time *t* since the beginning of the measurement, i.e. an ensemble average. Alternatively, it is possible to perform the average over all the displacements in a lag time Δ of a single trajectory, i.e. a temporal average. For ergodic systems, both averages converge to the same value. However, weak ergodicity breaking can take place as a consequence of kinetics with power-law statistics in the plasma membrane^36^,^37^ and in the cytoplasm of live cells^16^,^38^ as well as in inorganic complex systems such as quantum dots^39^,^40^ and models of glassy dynamics^33^.

## Results

### Diffusion of membrane-targeting proteins on supported lipid bilayers

We tracked the motion of the membrane-targeting C2A domain from synaptotagmin 7^41^, labelled with Atto-565, on a supported lipid bilayer composed of phosphatidylcholine (PC) and phosphatidylserine (PS) at a 3:1 ratio. The lipid bilayer was self-assembled on a clean coverslip^6^. Imaging was done in a home-built total internal reflection (TIRF) microscope under continuous illumination at 20 frames/s. Surface densities were kept low enough to enable accurate tracing of trajectories and to allow assignment of connections even after micrometer-long jumps. Fluorescence imaging revealed the surface density was 0.017 ± 0.005 *μm*^−2^ (mean ± standard deviation). The sudden appearance of fluorescent molecules in the TIRF field also provided an estimate of the adsorption rate *k*_*on*_. By counting the number of arrivals, we measured *k*_*on*_ = (2 ± 1) × 10^−4^ *s*^−1^*μm*^−2^.

Figure 2a shows an example of trajectories obtained in a 10-s window, overlaid on the last frame. Often, long jumps are observed in the particle trajectories as seen in the examples in Fig. 2b,c. These jumps suggest the C2A molecules detach from the surface and readsorb after brief excursions into the liquid bulk. The motion in the bulk is much faster than diffusion on the viscous membrane and jumps are thus expected to occur instantaneously for all practical purposes. For the C2A domain, the diffusion coefficient in the lipid bilayer *D*_*s*_ is of the order of 2 *μ*m^2^/s, but in liquid the diffusion coefficient *D*_*b*_ is estimated to be 100 times higher^42^. As a consequence, when a molecule performs a jump through the bulk it can sometimes be observed at reduced intensity in both the old and new locations within the same imaging frame, as seen in Fig. 2b. Given the measured *k*_*on*_, the probability that we misinterpret a new molecule being absorbed from the bulk, as a jump within 100 ms and a 3-*μ*m radius from a molecule that left the surface is only 6 × 10^−4^.

In order to study the effect of the dissociation constant, we also employed a C2A construct fused to a non-membrane interacting glutathione S-transferase (GST) that has a strong tendency to dimerize (Fig. 3a). The GST-C2A dimer forms two independent interactions with the membrane and will consequently have a slower dissociation rate than C2A monomer, providing a good comparison for validating our superdiffusion predictions. Additionally, GST-C2A dimer has a higher viscous drag coefficient and, in turn, its diffusion coefficient on the membrane surface is reduced to nearly half^43^. In a similar way as performed for C2A molecules, we estimated *k*_*on*_ = (1.0 ± 0.3) × 10^−4^ *s*^−1^*μm*^−2^.

We collected 14,000 C2A and 3,600 GST-C2A mobile trajectories. Immobile fluorophores that did not exhibit any apparent diffusive motion were excluded from the analysis. The ensemble-averaged MSD 〈*r*^2^(*t*)〉 of C2A monomers and dimer-forming GST-C2A are shown in Fig. 3b. In order to compute the ensemble-averaged MSD, a single displacement from each trajectory is employed. A deviation from a linear MSD is evident in the figure, showing superdiffusive behaviour. Further, the onset of superdiffusion for GST-C2A occurs at a later stage.

The time-averaged MSD 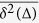 is often used in the analysis of individual trajectories. Throughout this manuscript we will denote the ensemble average of an observable with brackets 〈⋅〉 and the time average with an overbar.-. For a trajectory with *N* time points,


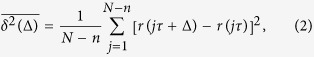


where *τ* is the time interval between consecutive measurements and *n* = Δ/*τ*. This approach is especially useful when a limited number of trajectories is available, as usually occurs in single-molecule studies. Figure 3c shows the time-averaged MSD after it is additionally averaged over all the trajectories. GST-C2A exhibits the expected slower diffusion rate than C2A, based on the MSD slope. As mentioned above, for ergodic processes, the temporal and ensemble averages coincide in the long time limit, 
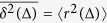
. However, the ergodic hypothesis breaks down for C2A molecules. In contrast to the ensemble-averaged MSD, the time-averaged MSD is linear in lag time


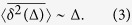


Thus, an observer analysing time-averages would reach the misleading conclusion that the diffusion behaviour is not anomalous.

The distribution of displacements *P*(**r**) at Δ = 100 ms is shown in Fig. 3d,e for C2A and GST-C2A, respectively. The distribution exhibits two different characteristic regimes: a central part up to a distance *r* ≈ 1.5 *μm* and a long tail. This behaviour can be understood from the scaling properties of bulk-mediated diffusion as discussed by Bychuk and O’Shaughnessy^44^. Once a molecule dissociates from the surface, it performs a three-dimensional random walk until it returns. In the asymptotic limit, the first return time distribution scales as *ψ*(*τ*) ~ *τ*^−1.5^. For any given return time, the surface distance between the dissociation and return points has a Gaussian distribution 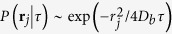. Therefore, the distribution of jump lengths is 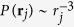, as observed in Fig. 3d,e for long distances.

The theoretical probability density function of jump lengths can be found using the image method^45^. The distance of first return to the surface are governed by 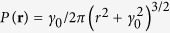, that is a two-dimensional Cauchy distribution. At short times, the probability that the particle performs more than a single jump is small. If we neglect the distance covered by surface diffusion within time intervals at which the particle undergoes a bulk excursion, the motion at each short interval is either by surface diffusion or via a jump. We can then approximate the distribution of displacements at short times by


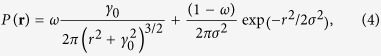


where *ω* is the probability that the particle hops within the given time and surface diffusion yields *σ*^2^ = 2*D*_*s*_*t*. A least-square fitting of the distribution of displacements (Fig. 3d,e) to this propagator yields *D*_*s*_ = 1.7 *μ*m^2^/s for C2A monomers and *D*_*s*_ = 1.0 *μ*m^2^/s for GST-C2A dimers. The parameter *γ* is found to be 0.24 *μ*m and 0.12 *μ*m for C2A and GST-C2A, respectively.

The distribution of displacements for longer times involves both a random number of jumps, each having a Cauchy distribution, and the Brownian motion on the surface. Chechkin *et al.* derived the full solution for the propagator of bulk-mediated diffusion^46^. For the case when *D*_*s*_ = 0 and neglecting long distance corrections, the distribution of displacements is given by the Cauchy propagator, in agreement with scaling arguments^44^,


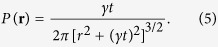


When the particles also diffuse on the surface, i.e. *D*_*s*_ ≠ 0 the probability density of the displacements is given by the convolution of equation (5) with a normal distribution. Even though the full solution for long times is complicated, the tail of this distribution for large distances still scales as *P*(**r**) ~ *r*^−3^. Due to this asymptotic behaviour, the exact distribution has similar properties to the Cauchy distribution.

### Numerical simulations: diffusion in the presence of bulk excursions

**Figure 1:**
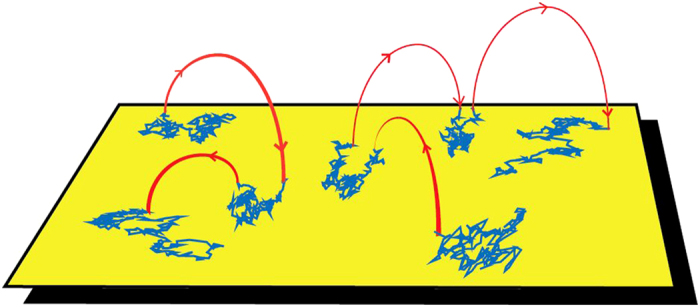
Sketch of the diffusion process. A molecule alternates between phases of two-dimensional and three-dimensional diffusion. Diffusion in the three-dimensional bulk is much faster than diffusion on the lipid bilayer, and thus only the effective two-dimensional process is observed without loss of trajectory connectivity. The excursions into the bulk are seen as long jumps in the two-dimensional trajectories.

**Figure 2:**
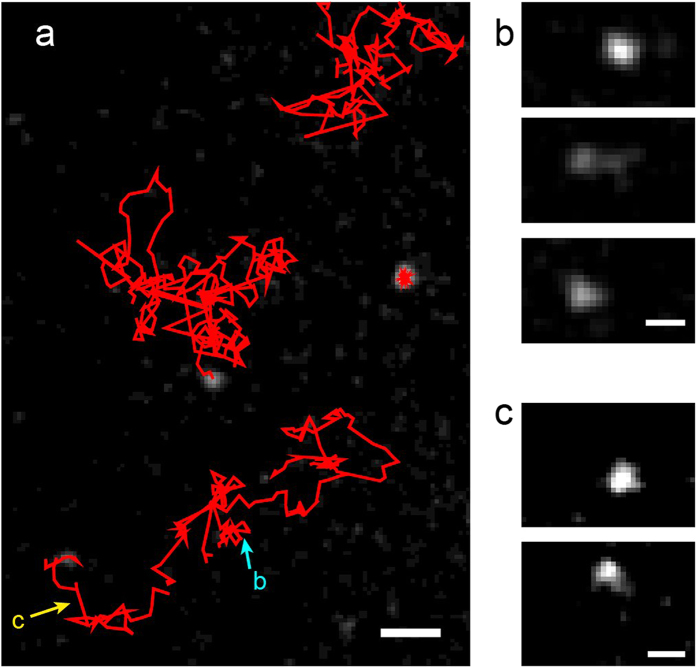
Single particle tracking of membrane-targeting domains. (**a**) C2A-Atto565 individual trajectories collected during a 10-s time window. Three mobile trajectories are observed in the image together with one immobile particle that is tracked but is not included in the analysis. The last frame is overlaid on the trajectories. The original data is shown in Supplementary Video S1. Scale bar 2 *μ*m. (**b**) Region of interest (ROI) around the location of a micrometer jump that occurs in the lowermost trajectory, marked with the letter b. Three frames are shown corresponding to before, during, and after the jump. Scale bar 0.5 *μ*m. (**c**) ROI around the location of the jump marked with the letter c. Scale bar 1 *μ*m.

In order to verify the model of surface diffusion in the presence of bulk excursions we analyse numerical simulations of the process diagrammed in Fig. 1. Molecules perform a two-dimensional random walk, but at random times they jump due to a hypothetical bulk excursion. The surface residence times are assumed to be independent and identically distributed exponential random variables and the jumps are modelled according to the first return time to the surface given simple diffusion in a three-dimensional medium. These simulations are analysed in the same way as with experimental observations of the motion of membrane-targeting C2 domains on supported membranes. 500 realizations were simulated off-lattice with a surface diffusion coefficient *D*_*s*_ = 0.5 and a dissociation coefficient *k* = 0.1. The chosen parameters do not intend to capture the real protein properties, but to simply test theoretical predictions without the effects of experimental noise. The displacements for two-dimensional diffusion are drawn from a Gaussian distribution with variance 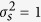 and the return times from bulk excursions are drawn from a distribution 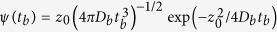
45. Then the jump distances are drawn from a Gaussian distribution with variance 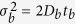.

The distribution of displacements *P*(**r**) for the numerical simulations is shown in Fig. 4a. As expected, there are two regimes: a central Gaussian part due to the two-dimensional diffusion on the membrane between bulk excursions, and a heavy tail that arises from the long distance behaviour of bulk excursions. The distributions for short times can again be modelled with a propagator that includes contributions from Gaussian surface diffusion and a Cauchy distribution due to bulk excursions. By fitting to equation (4), it is found *D*_*s*_ = 0.50 ± 0.05 (the value employed in the simulations is *D*_*s*_ = 0.5) and *γ*_0_ = 0.75.

### MSD analysis

The dynamics of a particle with a Cauchy propagator are particularly interesting because the theoretical variance of the displacements diverges,


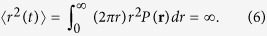


In practice, a diverging second moment implies that there is a non-negligible probability for the occurrence of extremely long jumps and this phenomenon has direct implications in the measured MSD. Figure 4b shows the ensemble-averaged MSD computed from the numerical simulations. The MSD increases in a superlinear fashion, i.e. by employing equation (1), we have *α* > 1, which implies the process is superdiffusive.

**Figure 3:**
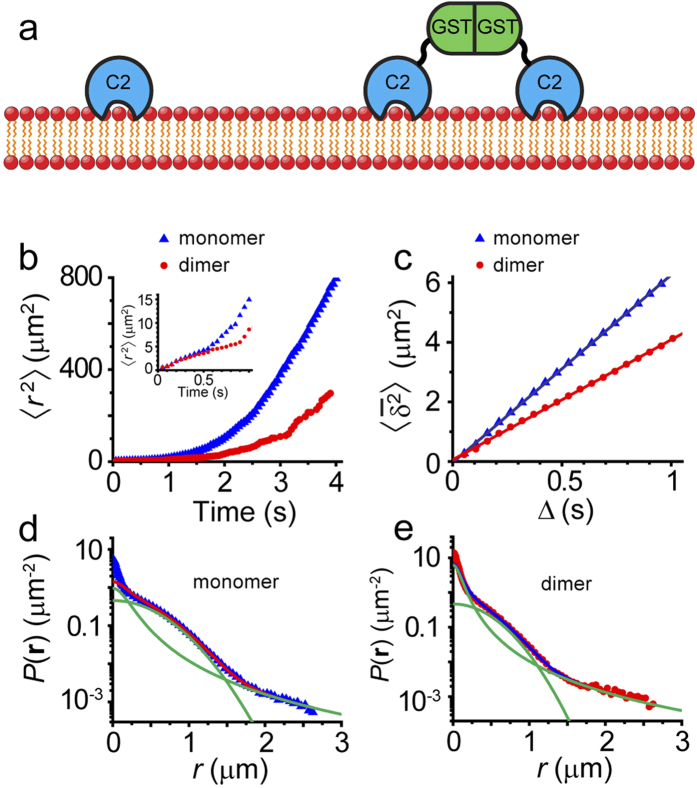
Anomalous diffusion analysis of membrane-targeting domain C2A (monomer) and dimer forming GST-C2A. (**a**) Sketch of the C2A monomer and the GST-C2A dimer employed in this study. (**b**) Ensemble-averaged MSD 〈*r*^2^(*t*)〉. The inset provides a zoom of the data so that it is presented with the same time axis as the time-averaged MSD, for comparison. (**c**) Time averaged MSD 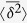 as a function of lag time Δ. The time-averaged MSD of individual trajectories varies greatly, so the MSDs of individual trajectories are also ensemble averaged. (**d**,**e**) Distribution of displacements for Δ = 100 ms. The total number of displacements are 207,000 and 56,000 for C2A and GST-C2A, respectively. The solid lines show fitting to equation (4) and to the individual components of the propagator, i.e. the Gaussian part [(1 − *ω*)/2*πσ*^2^]exp(−*r*^2^/2*σ*^2^) and the Cauchy propagator part *ωγ*/2*π*(*r*^2^ + *γ*^2^)^3/2^. The cutoff at 2.6 *μ*m appears because trajectories are not connected when jumps longer than this distance take place. This threshold is placed in order to avoid the risk of particle misconnections.

**Figure 4:**
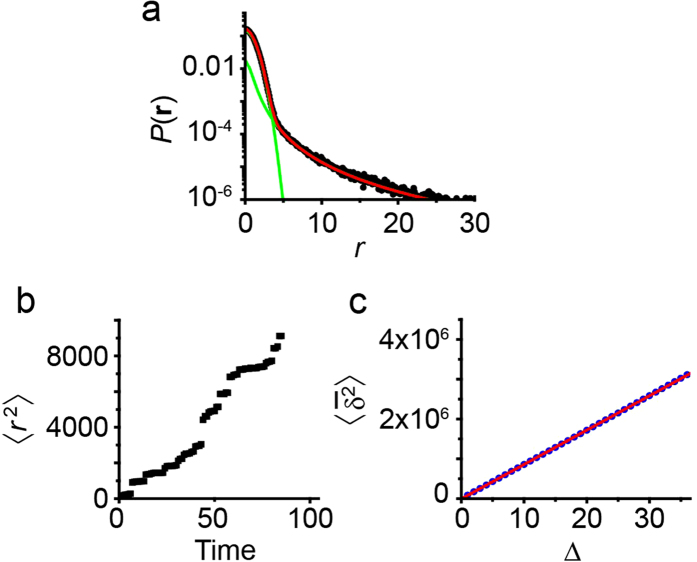
Numerical simulations of Lévy flights. 500 realizations were performed, in which a particle alternates between 2D random walks and bulk-mediated jumps. (**a**) Probability density of the tracer displacements. The density is well described by a theoretical model that includes a Gaussian central part and a Cauchy propagator of the form 
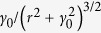
. (**b**) Ensemble-averaged MSD 〈*r*^2^(*t*)〉 as a function of time. The ensemble-averaged MSD is computed from the distance covered by the tracer in a time *t* from the start of the realization. (**c**) The time-averaged MSD 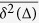 is averaged over all realizations and plot against lag time Δ.

Let us now analyse the unexpected MSD behaviour, starting from the time-averaged MSD of individual trajectories. We can show that the time-averaged MSD is linear in lag time for any random walk with independent increments **u**_*i*_ = **r**_*i*+1_ − **r**_*i*_, such that 〈**u**_*i*_ ⋅ **u**_*j*_〉 = 0 when *i* ≠ *j*. From the definition of the time-averaged MSD (equation (2))^47^,


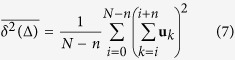



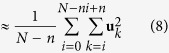



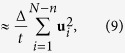


where we have used the approximation that *t* ≫ Δ, we omitted the term 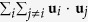 because it is zero on average, and again we have used the parameter *n* = Δ/*τ*. Therefore we see that for symmetric random walks with independent increments, the time-averaged MSD is linear as observed in Figs 3c and 4c.

Although the time-averaged MSD for individual trajectories is linear, the ensemble averaged MSD 〈*r*^2^(*t*)〉 is not. We can understand the superdiffusive behaviour by assuming we can define the motion in terms of two independent processes **r**(*t*) = ***b***(*t*) + **y**(*t*), where **b**(*t*) is a two-dimensional Brownian motion and **y**(*t*) is a Lévy process with a probability density defined by equation (5). Then the MSD is 〈**r**^2^〉 = 〈**b**^2^〉 + 〈**y**^2^〉. The first term is linear in time but the second term has a superdiffusive nature^44^,^46^,^48^.

## Discussion

The results presented in this article are due to bulk-mediated jumps. Pólya’s theorem states a random walk in one dimension will return to the origin with probability one^49^. However, we may ask how likely it is for a particle that dissociates from the surface to re-associate within a given measurement time. We can estimate this probability from the distribution of first passage times in one dimension^45^. Let us, for the sake of simplicity, consider that a molecule dissociates from the surface when it reaches a height *z*_0_ = 10 nm. Then, using a generic diffusion coefficient *D*_*b*_ = 100 *μm*^2^/s, we find the probability of not returning to the surface within less than 50 ms to be as low as 0.0025. This means that the vast majority of molecules dissociating from the surface return in very short times. In general, the probability of no return in time *τ* is 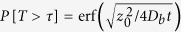.

The propagator for surface diffusion in the presence of bulk-mediated jumps (equation (4)) depends on the surface diffusion coefficient *D*_*s*_ and the parameter *γ* that reflects the transition between the surface and the bulk phase. Namely, *γ* ~ *a*/*τ*_des_, where *τ*_des_ is the mean desorption time and *a* is a dimensional factor. Bulk-mediated diffusion thus predicts *γ*_dimer_ < *γ*_monomer_, in agreement with the values we find for C2A and GST-C2A.

The surface motion of these membrane-targeting domains is well described by Lévy flights, a random walk where the step displacements have a heavy-tailed distribution. The heavy tail arises from the dissociation of molecules from the membrane, which then perform a three-dimensional random walk until they reach the surface again at another location. The process involves the first return to a surface and it converges to a power law according to the Sparre-Andersen theorem^45^. This type of Lévy flight dynamics is fundamentally different from Lévy walks induced by molecular motors in the cytoplasm because periods of active motion require an energy input, typically in the form of ATP hydrolysis, while bulk excursions occur spontaneously.

One of the most interesting and diagnostic effects of the observed bulk-mediated diffusion statistics is that the ensemble-averaged MSD exhibits superdiffusive behaviour, whereas the temporal averages suggest normal diffusion. This nonergodic behaviour is similar to that of continuous time random walks (CTRW) where the sojourn time distribution between steps has a probability distribution that is heavy-tailed. Also in the CTRW, 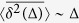 and 〈*r*^2^(*t*)〉 ~ *t*^*α*^, albeit the CTRW is subdiffusive with *α* < 1. The difference in the behaviour of temporal and ensemble averages is the key signature of weak ergodicity breaking in the process^50^.

To date, different groups have observed normal diffusion for membrane proteins in supported lipid bilayers, which appear to contradict our findings^51^,^52^,^53^,^54^. There are several reasons for this apparent discrepancy. Single-particle tracking in lipid bilayers often focuses on time-averaged MSD, which does not show any non-linearity in lag time. Thus it would be reasonable to reach the conclusion that diffusion is not anomalous. Furthermore, anomalous diffusion in supported bilayers is known to develop as a result of packing and crowding. These mechanisms are modelled by a fractional Langevin equation, which is ergodic in nature, with anomalies that show up in the time averages. The distribution of displacements has also been previously reported as exhibiting Gaussian behaviour. Here we report on the motion of surface-bound membrane domains that exhibit desorption from the membrane within the experimental observation time. Previous works dealing with membrane-targeting domains such as C2 have generally been limited to short displacements in order to exclude the effect of long bulk-mediated jumps in diffusion measurements^43^,^54^. The behaviour of trans- and integral membrane proteins or lipids is very different because the free energy barrier for release from the membrane is too high to be observed within the constraints of experimental observations^51^,^52^,^53^.

What are the biological implications of surface superdiffusion for peripheral membrane proteins? Search processes are ubiquitous in cell biology and it is feasible to assume that evolution has optimized search parameters. For signalling molecules delivered to the plasma membrane during a specific stimulus, the target molecule is often scarce in a sea of other lipids and proteins. Thus we can envision that if a molecule does not find its target in a given time, it becomes more efficient to start searching at a different location. But is it then appropriate to assume Lévy flights yield the optimal search for sparse targets when compared to Brownian motion? For one-dimensional intermittent processes that switch between Brownian motion and ballistic relocation phases, it has been shown that the search process is significantly more efficient when relocation times are power-law distributed, resulting in a Lévy walk^55^. Notably, when Lévy dynamics are employed, the search is less sensitive to the target density^55^. In general, the optimal strategy depends on the average target distance from the starting point^56^. However, blind searches inside a living cell are very different from a search in an unobstructed environment. Several aspects provide additional complexities, particularly in the plasma membrane^13^. Experimental measurements show that the plasma membrane is compartmentalized in a way that proteins and lipids have the tendency to remain transiently confined within small regions^57^. Further, membrane proteins typically exhibit subdiffusion with anti-persistent increments where molecules drift towards the locations that they visited in the past. While this subdiffusive behaviour provides the opportunity for a thorough and compact search, it is definitely not the optimum situation to find sparse targets. A superdiffusive Lévy flight provides a mechanism to overcome the effects of anti-persistent correlated subdiffusive motion. Thus, we expect Lévy flight dynamics to often outperform a Brownian search.

The obstruction to the diffusion of membrane molecules has two different sources, both of them causing anti-persistent correlations in the random walk. On one hand, obstacles can be introduced by immobile transmembrane proteins which affect all lipids and membrane proteins. On the other hand, a more severe obstruction can be caused by cytoskeleton components that may not be in direct contact with the plasma membrane^58^. The effect of these barriers is not equal for all membrane proteins. Proteins that have large intracellular complexes are blocked much more efficiently than small molecules. In cases where a large signalling molecule adheres to the membrane via phospholipid-binding domains, bulk excursions allow for the exploration of larger areas. Otherwise, the molecule would remain confined for long times within cytoskeleton-formed corrals, even when no substrate target is found within this region.

In summary, we have observed the nonergodic, superdiffusive motion of membrane-targeting peptide domains in supported lipid bilayers. The motion is well-described by Lévy flights with jumps that have a heavy-tail distribution. The long jumps are caused by excursions into the liquid bulk. After dissociating from the membrane, the molecules diffuse in three dimensions until they reach the membrane again and bind at a new location. Diffusion in the liquid bulk is much faster than diffusion in the membrane, therefore we do not consider the delay time between dissociation and readsorption. The surface distances covered by jumps have a Cauchy distribution, which is responsible for the heavy tail in the superdiffusive Lévy flights. Model membranes provide an elegant system to study the effect of superdiffusive Lévy flights because they are not subjected to the interactions with other cell components that would mask its experimental observation. However, hopping was already observed on the surface of live cells^7^ and we foresee these processes have broad physiological relevance in the surface diffusion of signalling molecules.

## Methods

### Imaging buffer

Imaging and rinsing during the preparation steps was performed in an imaging buffer consisting of 50 mM HEPES, 75 mM NaCl, 1 mM MgCl_2_, 2 mM tris(2-carboxyethyl)phosphine (TCEP), 200 *μ*M CaCl_2_. CaCl_2_ is necessary for C2 domain binding to the reconstituted membrane.

### Preparation of phospholipid vesicles

Phospholipids were purchased from Avanti Polar Lipids (Alabaster, AL). Chloroform-suspended 18:1 (Δ9-Cis) PC (DOPC) and 18:1 PS (DOPS) were mixed at a ratio of 3:1. The phospholipid mixture was vacuum dried overnight and resuspended in imaging buffer to a final concentration of 3 mM followed by probe sonication to form sonicated unilamellar vesicles (SUVs).

### Preparation of coverslips and supported lipid bilayers

Glass coverslips were cleaned by sonication in a detergent solution followed by soaking in 1M KOH. The coverslips were rinsed extensively in Milli-Q water and blown dry with a stream of nitrogen gas. Then, the coverslips were treated with an oxygen plasma. Immediately after the plasma cleaning, a perfusion chamber (CoverWell, Grace Bio-Labs model PC8R-1.0) was adhered to the coverslip. In order to deposit the lipid bilayers, a solution of SUVs (1.5-mM lipid) composed of phosphatidylcholine (PC) and phosphatidylserine (PS) at a 3:1 ratio in 0.5 M NaCl and imaging buffer was introduced into the perfusion chamber and incubated for one hour at 4 °C. Refrigeration minimizes lipid oxidation. The surface was then rinsed with imaging buffer multiple times prior to addition of protein sample.

### C2A and GST-C2A expression and purification

An expression plasmid containing the gene for a GST-ybbR-Synaptotagmin 7 (Syt7) C2A domain fusion protein was transformed into *E. coli* BL21-CodonPlus(DE3) competent cells. The ybbR segment provides a site for Sfp-catalysed fluorophore labelling^59^. Cells were grown at 37 °C to an OD_600_ of 0.6 and then induced to express protein with 0.5 mM IPTG at room temperature for 6 hours. The harvested cells were lysed at 18,000 lb/in^2^ in a microfluidizer in a buffer containing 50 mM Tris pH 7.5, 400 mM NaCl and centrifuged at 17,000 rpm in a Sorval SS-34 rotor. The clarified lysate was loaded onto a 5-ml GSTrap FF column (GE Healthcare LifeSciences, Pittsburgh, PA) followed by gradient elution with 50 mM Tris, pH 8.0, 100 mM NaCl, and 10 mM glutathione. Fractions containing protein were pooled and diluted to reduce the salt to less than 0.1 M prior to loading onto a HiTrap Q HP column (GE Healthcare LifeSciences, Pittsburgh, PA) and eluting with a linear gradient to 1 M NaCl in 25 mM Tris, pH 8.5, 20%(vol/vol) glycerol, and 0.02%(wt/vol) NaN_3_. A portion of the construct was subjected to thrombin cleavage and then separated using a Superdex 200 gel filtration column (GE Healthcare LifeSciences, Pittsburgh, PA) equilibrated in 50 mM Tris, pH 7.5 and 100 mM NaCl to yield a ybbr-Syt7 C2A construct.

### Protein labelling

20 mM CoASH (New England Biolabs, Ipswich, MA) in 400 mM Tris, pH 7.5 was mixed with 20 mM ATTO-565 maleimide (ATTO-TEC, Siegen, Germany) in dimethylformamide and incubated at 30 °C overnight to form ATTO-565 CoA, then quenched by 10-fold dilution into 5 mM DTT, 10 mM Tris pH 7.5. 10 *μ*M GST-ybbr-Syt7 C2A and ybbr-Syt7 C2A were labelled with the ATTO-565 via SFP synthase (4′-phosphopantetheinyl transferase). Each reaction contained 50 mM Tris 7.5, 10 mM MgCl_2_, 40 mM NaCl, 20 *μ*M ATTO-565 CoA and 1 *μ*M SFP synthase. Reactions were incubated at room temperature for 30 minutes, then placed at 4 °C overnight. Samples were dialysed against 1 L of 50 mM HEPES, pH 7.0, 75 mM NaCl, 4 mM MgCl_2_ and 5% glycerol overnight at 4 °C then concentrated to 10 *μ*M.

### Imaging

Proteins were added to the imaging buffer to a final concentration of 75 pM. Then, the perfusion chamber was filled with the solution and after a short incubation period of 10 minutes we started imaging. The employed perfusion chambers were 9 mm in diameter and 0.9-mm deep, holding a volume of ≈60 *μ*l. Imaging was performed without replacing the solution, so that there was always protein present in the bulk solution and the surface concentration could reach a steady state.

All images were acquired using an objective-type total internal reflection fluorescence microscope (TIRFM). The microscope was home-built around an Olympus IX71 body^18^,^36^ with a 561 nm laser line as excitation source. A back-illuminated electron-multiplied charge coupled device (EMCCD) camera (Andor iXon DU-888) liquid-cooled to −85 °C, with an electronic gain of 300 was used. In order to maintain constant focus during the whole imaging time we employed an autofocus system (CRISP, Applied Scientific Instrumentation, Eugene, OR) in combination with a piezoelectric stage (Z-100, Mad City Labs, Madison, WI). Videos were acquired at a frame rate of 20 frames/s.

### Image processing and single-particle tracking

Images were acquired using Andor IQ 2.3 software and saved as 16-bit tiff files. Then the images were filtered using a Gaussian kernel with a standard deviation of 1.0 pixel in ImageJ. Single-particle tracking of Atto-C2 and Atto-GST-C2 was performed in MATLAB using the U-track algorithm developed by Jaqaman *et al.*^60^ under thorough manual inspection of detection and tracking.

## Additional Information

**How to cite this article**: Campagnola, G. *et al.* Superdiffusive motion of membrane-targeting C2 domains. *Sci. Rep.* **5**, 17721; doi: 10.1038/srep17721 (2015).

## Supplementary Material

Supplementary Video S1
